# 4-(1,3-Benzothia­zol-2-yl)-1,5-dimethyl-2-phenyl-1*H*-pyrazol-3(2*H*)-one

**DOI:** 10.1107/S1600536811037652

**Published:** 2011-09-30

**Authors:** Imane Chakib, Abdelfettah Zerzouf, Youssef Kandri Rodi, El Mokhtar Essassi, Seik Weng Ng

**Affiliations:** a Laboratoire de Chimie Organique Hétérocyclique, Pôle de Compétences Pharmacochimie, Université Mohammed V-Agdal, BP 1014 Avenue Ibn Batout, Rabat, Morocco; b Laboratoire de Chimie Organique Appliquée, Faculté des Sciences et Techniques, Université Sidi Mohamed Ben Abdallah, Fés, Morocco; c Department of Chemistry, University of Malaya, 50603 Kuala Lumpur, Malaysia; d Chemistry Department, Faculty of Science, King Abdulaziz University, PO Box 80203 Jeddah, Saudi Arabia

## Abstract

The central five-membered ring of the title compound, C_18_H_15_N_3_OS, is almost planar (r.m.s. deviation = 0.028 Å) and the benzothia­zole fused-ring system is close to coplanar with this ring [dihedral angle = 6.1 (1)°]. The phenyl substituent is twisted by 62.5 (1)°.

## Related literature

For the structure of the reactant 4-(2,3-dihydro-1,3-benzothiazol-2-ylidene)-3-methyl-1-phenyl-1*H*-pyrazol-5(4*H*)-one, see: Chakibe *et al.* (2010 ▶).
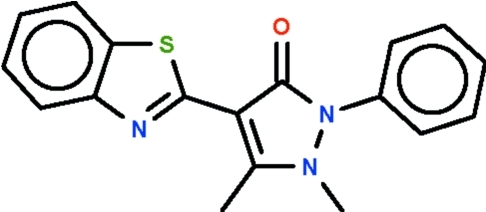


## Experimental

### 

#### Crystal data

C_18_H_15_N_3_OS
*M* *_r_* = 321.39
Monoclinic, 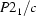

*a* = 8.7428 (2) Å
*b* = 25.7551 (5) Å
*c* = 6.9660 (1) Å
β = 97.460 (1)°
*V* = 1555.27 (5) Å^3^
*Z* = 4
Mo *K*α radiation
μ = 0.22 mm^−1^
*T* = 293 K
0.50 × 0.10 × 0.10 mm

#### Data collection

Bruker APEXII diffractometer
Absorption correction: multi-scan (*SADABS*; Sheldrick, 1996 ▶) *T*_min_ = 0.900, *T*_max_ = 0.979
18953 measured reflections
3569 independent reflections
2418 reflections with *I* > 2σ(*I*)
*R*_int_ = 0.053

#### Refinement

*R*[*F* ^2^ > 2σ(*F* ^2^)] = 0.047
*wR*(*F* ^2^) = 0.131
*S* = 1.01
3569 reflections
210 parameters
H-atom parameters constrained
Δρ_max_ = 0.26 e Å^−3^
Δρ_min_ = −0.27 e Å^−3^

### 

Data collection: *APEX2* (Bruker, 2005 ▶); cell refinement: *SAINT* (Bruker, 2005 ▶); data reduction: *SAINT*; program(s) used to solve structure: *SHELXS97* (Sheldrick, 2008 ▶); program(s) used to refine structure: *SHELXL97* (Sheldrick, 2008 ▶); molecular graphics: *X-SEED* (Barbour, 2001 ▶); software used to prepare material for publication: *publCIF* (Westrip, 2010 ▶).

## Supplementary Material

Crystal structure: contains datablock(s) global, I. DOI: 10.1107/S1600536811037652/jh2331sup1.cif

Structure factors: contains datablock(s) I. DOI: 10.1107/S1600536811037652/jh2331Isup2.hkl

Supplementary material file. DOI: 10.1107/S1600536811037652/jh2331Isup3.cml

Additional supplementary materials:  crystallographic information; 3D view; checkCIF report

## Figures and Tables

**Fig. 1.**
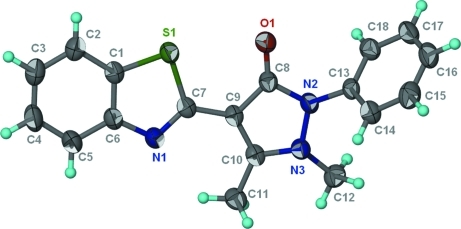
Thermal ellipsoid plot (Barbour, 2001) of C18H15N3OS at the 50% probability level; hydrogen atoms are drawn as spheres of arbitrary radius.

## supplementary crystallographic information

### Comment

In the study, the tertiary nitrogen atom of the five-membered ring of 4-(2,3-dihydro-1,3-benzothiazol-2-ylidene)-3-methyl-1-phenyl-1*H*-pyrazol-5(4*H*)-one (Chakibe *et al.*, 2010) is used to displace iodine from methyl iode to give the title compound; the carbon-carbon double-bond in the reactant is consequently converted to a double bond (Scheme I, Fig. 1). The central five-membered ring and the benzothiazolyl fused-ring is nearly co-planar (dihedral angle 6.1 (1) °). The phenyl substituent is twisted by 62.5 (1) ° with respect to the five-membered ring.

### Experimental

To a solution of (*E*)-4-(2,3-dihydro-1,3-benzothiazol-2-ylidene)-3-methyl-1-phenyl-1*H*-pyrazol-5(4*H*)-one (1 g, 3.25 mmol) in DMF (50 ml) was added sodium carbonate (2.5 g, 23 mmol), tetra-*n*-butylammonium bromide (0.15 g, 1 mmol) and methyl iodide (7.1 g, 50 mmol). The mixture was stirred for 24 h. The solid material was removed b filtration and the solution was evaporated. The residue was washed with dichloromethane and hexane, and was recrystallized from ethanol to afford the title compound as colorless crystals.

### Refinement

Carbon-bound H-atoms were placed in calculated positions (C—H 0.93–0.96 Å) and were included in the refinement in the riding model approximation, with *U*(H) set to 1.2–1.5*U*(C). Omitted from the refinement was the (0 2 0) reflection.

### Crystal data

**Table:**

C_18_H_15_N_3_OS	*F*(000) = 672
*M**_r_* = 321.39	*D*_x_ = 1.373 Mg m^−^^3^
Monoclinic, *P*2_1_/*c*	Mo *K*α radiation, λ = 0.71073 Å
Hall symbol: -P 2ybc	Cell parameters from 3894 reflections
*a* = 8.7428 (2) Å	θ = 2.5–24.6°
*b* = 25.7551 (5) Å	µ = 0.22 mm^−^^1^
*c* = 6.9660 (1) Å	*T* = 293 K
β = 97.460 (1)°	Prism, colorless
*V* = 1555.27 (5) Å^3^	0.50 × 0.10 × 0.10 mm
*Z* = 4	

### Data collection

**Table:**

Bruker APEXII diffractometer	3569 independent reflections
Radiation source: fine-focus sealed tube	2418 reflections with *I* > 2σ(*I*)
graphite	*R*_int_ = 0.053
φ and ω scans	θ_max_ = 27.5°, θ_min_ = 2.4°
Absorption correction: multi-scan (*SADABS*; Sheldrick, 1996)	*h* = −11→11
*T*_min_ = 0.900, *T*_max_ = 0.979	*k* = −29→33
18953 measured reflections	*l* = −9→9

### Refinement

**Table:**

Refinement on *F*^2^	Primary atom site location: structure-invariant direct methods
Least-squares matrix: full	Secondary atom site location: difference Fourier map
*R*[*F*^2^ > 2σ(*F*^2^)] = 0.047	Hydrogen site location: inferred from neighbouring sites
*wR*(*F*^2^) = 0.131	H-atom parameters constrained
*S* = 1.01	*w* = 1/[σ^2^(*F*_o_^2^) + (0.0644*P*)^2^ + 0.296*P*] where *P* = (*F*_o_^2^ + 2*F*_c_^2^)/3
3569 reflections	(Δ/σ)_max_ = 0.001
210 parameters	Δρ_max_ = 0.26 e Å^−^^3^
0 restraints	Δρ_min_ = −0.27 e Å^−^^3^

### Fractional atomic coordinates and isotropic or equivalent isotropic displacement parameters (Å^2^)

**Table:**

	*x*	*y*	*z*	*U*_iso_*/*U*_eq_	
S1	0.92125 (7)	0.46686 (2)	0.19736 (8)	0.04602 (18)	
N1	0.8107 (2)	0.43170 (7)	0.5013 (3)	0.0485 (5)	
N2	0.6696 (2)	0.60559 (6)	0.3813 (2)	0.0450 (4)	
N3	0.6087 (2)	0.58549 (6)	0.5419 (2)	0.0436 (4)	
O1	0.80712 (19)	0.57089 (6)	0.1499 (2)	0.0555 (4)	
C1	0.9552 (2)	0.40265 (8)	0.2603 (3)	0.0435 (5)	
C2	1.0365 (3)	0.36554 (8)	0.1694 (3)	0.0531 (6)	
H2	1.0832	0.3740	0.0609	0.064*	
C3	1.0459 (3)	0.31630 (9)	0.2442 (4)	0.0611 (7)	
H3	1.1003	0.2910	0.1862	0.073*	
C4	0.9756 (3)	0.30371 (9)	0.4045 (4)	0.0682 (7)	
H4	0.9818	0.2698	0.4510	0.082*	
C5	0.8967 (3)	0.34025 (9)	0.4969 (4)	0.0657 (7)	
H5	0.8506	0.3313	0.6053	0.079*	
C6	0.8869 (2)	0.39090 (8)	0.4253 (3)	0.0462 (5)	
C7	0.8201 (2)	0.47347 (7)	0.3978 (3)	0.0399 (5)	
C8	0.7531 (2)	0.56620 (8)	0.3030 (3)	0.0417 (5)	
C9	0.7516 (2)	0.52318 (7)	0.4329 (3)	0.0387 (5)	
C10	0.6676 (2)	0.53751 (7)	0.5781 (3)	0.0398 (5)	
C11	0.6421 (3)	0.50940 (9)	0.7570 (3)	0.0514 (6)	
H11A	0.5334	0.5064	0.7630	0.077*	
H11B	0.6897	0.5282	0.8679	0.077*	
H11C	0.6868	0.4754	0.7559	0.077*	
C12	0.5534 (3)	0.62109 (9)	0.6788 (3)	0.0527 (6)	
H12A	0.5437	0.6031	0.7972	0.079*	
H12B	0.4546	0.6346	0.6253	0.079*	
H12C	0.6252	0.6492	0.7045	0.079*	
C13	0.5892 (3)	0.64466 (7)	0.2625 (3)	0.0414 (5)	
C14	0.4314 (3)	0.64253 (9)	0.2115 (3)	0.0532 (6)	
H14	0.3732	0.6167	0.2606	0.064*	
C15	0.3615 (3)	0.67960 (10)	0.0859 (4)	0.0657 (7)	
H15	0.2553	0.6787	0.0497	0.079*	
C16	0.4484 (4)	0.71801 (10)	0.0139 (4)	0.0673 (8)	
H16	0.4007	0.7428	−0.0705	0.081*	
C17	0.6046 (4)	0.71958 (9)	0.0666 (4)	0.0627 (7)	
H17	0.6629	0.7454	0.0175	0.075*	
C18	0.6759 (3)	0.68310 (8)	0.1916 (3)	0.0508 (5)	
H18	0.7820	0.6844	0.2281	0.061*	

### Atomic displacement parameters (Å^2^)

**Table:**

	*U*^11^	*U*^22^	*U*^33^	*U*^12^	*U*^13^	*U*^23^
S1	0.0543 (3)	0.0407 (3)	0.0427 (3)	0.0044 (2)	0.0050 (2)	0.0038 (2)
N1	0.0525 (11)	0.0414 (10)	0.0524 (11)	0.0046 (8)	0.0100 (9)	0.0068 (8)
N2	0.0576 (11)	0.0379 (9)	0.0397 (9)	0.0063 (8)	0.0079 (8)	0.0048 (7)
N3	0.0568 (11)	0.0403 (10)	0.0334 (9)	0.0041 (8)	0.0047 (8)	−0.0002 (7)
O1	0.0732 (11)	0.0503 (9)	0.0456 (9)	0.0109 (8)	0.0180 (8)	0.0067 (7)
C1	0.0397 (11)	0.0404 (11)	0.0480 (12)	−0.0006 (9)	−0.0033 (9)	0.0008 (9)
C2	0.0500 (13)	0.0493 (13)	0.0590 (14)	0.0057 (11)	0.0036 (11)	−0.0051 (11)
C3	0.0572 (15)	0.0471 (14)	0.0778 (17)	0.0078 (11)	0.0044 (13)	−0.0065 (12)
C4	0.0668 (17)	0.0390 (13)	0.099 (2)	0.0085 (12)	0.0103 (15)	0.0110 (13)
C5	0.0698 (16)	0.0469 (14)	0.0841 (18)	0.0074 (12)	0.0240 (14)	0.0183 (13)
C6	0.0429 (12)	0.0386 (11)	0.0560 (13)	0.0005 (9)	0.0026 (10)	0.0061 (9)
C7	0.0398 (11)	0.0391 (11)	0.0383 (10)	−0.0017 (9)	−0.0043 (8)	0.0018 (8)
C8	0.0485 (12)	0.0376 (11)	0.0381 (11)	0.0028 (9)	0.0016 (9)	−0.0011 (8)
C9	0.0424 (11)	0.0360 (10)	0.0353 (10)	0.0003 (9)	−0.0036 (8)	−0.0004 (8)
C10	0.0433 (11)	0.0389 (11)	0.0342 (10)	−0.0014 (9)	−0.0067 (8)	−0.0005 (8)
C11	0.0613 (14)	0.0524 (13)	0.0392 (11)	0.0003 (11)	0.0020 (10)	0.0049 (10)
C12	0.0629 (15)	0.0518 (13)	0.0438 (12)	0.0052 (11)	0.0077 (11)	−0.0078 (10)
C13	0.0561 (13)	0.0316 (10)	0.0365 (10)	0.0055 (9)	0.0052 (9)	−0.0033 (8)
C14	0.0567 (14)	0.0439 (12)	0.0582 (14)	0.0007 (11)	0.0043 (11)	0.0035 (10)
C15	0.0643 (16)	0.0681 (17)	0.0617 (16)	0.0190 (13)	−0.0025 (13)	0.0022 (13)
C16	0.098 (2)	0.0560 (15)	0.0487 (14)	0.0268 (15)	0.0123 (14)	0.0137 (11)
C17	0.094 (2)	0.0384 (13)	0.0596 (15)	0.0055 (13)	0.0241 (14)	0.0075 (11)
C18	0.0626 (14)	0.0399 (12)	0.0506 (13)	−0.0025 (11)	0.0100 (11)	−0.0024 (10)

### Geometric parameters (Å, °)

**Table:**

S1—C1	1.727 (2)	C8—C9	1.432 (3)
S1—C7	1.755 (2)	C9—C10	1.376 (3)
N1—C7	1.304 (2)	C10—C11	1.483 (3)
N1—C6	1.386 (3)	C11—H11A	0.9600
N2—C8	1.401 (3)	C11—H11B	0.9600
N2—N3	1.399 (2)	C11—H11C	0.9600
N2—C13	1.428 (2)	C12—H12A	0.9600
N3—C10	1.350 (2)	C12—H12B	0.9600
N3—C12	1.451 (3)	C12—H12C	0.9600
O1—C8	1.227 (2)	C13—C18	1.377 (3)
C1—C2	1.392 (3)	C13—C14	1.381 (3)
C1—C6	1.395 (3)	C14—C15	1.382 (3)
C2—C3	1.369 (3)	C14—H14	0.9300
C2—H2	0.9300	C15—C16	1.381 (4)
C3—C4	1.382 (4)	C15—H15	0.9300
C3—H3	0.9300	C16—C17	1.368 (4)
C4—C5	1.375 (4)	C16—H16	0.9300
C4—H4	0.9300	C17—C18	1.374 (3)
C5—C6	1.395 (3)	C17—H17	0.9300
C5—H5	0.9300	C18—H18	0.9300
C7—C9	1.448 (3)		
			
C1—S1—C7	88.78 (10)	C8—C9—C7	122.65 (19)
C7—N1—C6	110.27 (18)	N3—C10—C9	109.58 (17)
C8—N2—N3	108.35 (15)	N3—C10—C11	120.60 (19)
C8—N2—C13	121.79 (16)	C9—C10—C11	129.77 (19)
N3—N2—C13	120.94 (17)	C10—C11—H11A	109.5
C10—N3—N2	108.24 (16)	C10—C11—H11B	109.5
C10—N3—C12	127.40 (17)	H11A—C11—H11B	109.5
N2—N3—C12	119.07 (16)	C10—C11—H11C	109.5
C2—C1—C6	121.6 (2)	H11A—C11—H11C	109.5
C2—C1—S1	128.56 (18)	H11B—C11—H11C	109.5
C6—C1—S1	109.83 (16)	N3—C12—H12A	109.5
C3—C2—C1	118.1 (2)	N3—C12—H12B	109.5
C3—C2—H2	120.9	H12A—C12—H12B	109.5
C1—C2—H2	120.9	N3—C12—H12C	109.5
C2—C3—C4	120.9 (2)	H12A—C12—H12C	109.5
C2—C3—H3	119.5	H12B—C12—H12C	109.5
C4—C3—H3	119.5	C18—C13—C14	121.0 (2)
C5—C4—C3	121.4 (2)	C18—C13—N2	117.5 (2)
C5—C4—H4	119.3	C14—C13—N2	121.43 (19)
C3—C4—H4	119.3	C15—C14—C13	118.7 (2)
C4—C5—C6	119.0 (2)	C15—C14—H14	120.7
C4—C5—H5	120.5	C13—C14—H14	120.7
C6—C5—H5	120.5	C14—C15—C16	120.4 (3)
N1—C6—C5	125.6 (2)	C14—C15—H15	119.8
N1—C6—C1	115.37 (18)	C16—C15—H15	119.8
C5—C6—C1	119.0 (2)	C17—C16—C15	120.1 (2)
N1—C7—C9	125.38 (19)	C17—C16—H16	120.0
N1—C7—S1	115.75 (15)	C15—C16—H16	120.0
C9—C7—S1	118.85 (15)	C16—C17—C18	120.3 (2)
O1—C8—N2	123.08 (18)	C16—C17—H17	119.9
O1—C8—C9	131.43 (19)	C18—C17—H17	119.9
N2—C8—C9	105.45 (17)	C17—C18—C13	119.6 (2)
C10—C9—C8	107.86 (17)	C17—C18—H18	120.2
C10—C9—C7	129.34 (18)	C13—C18—H18	120.2
			
C8—N2—N3—C10	7.4 (2)	N2—C8—C9—C10	1.0 (2)
C13—N2—N3—C10	155.18 (17)	O1—C8—C9—C7	−0.5 (4)
C8—N2—N3—C12	163.59 (18)	N2—C8—C9—C7	176.98 (18)
C13—N2—N3—C12	−48.6 (3)	N1—C7—C9—C10	1.4 (3)
C7—S1—C1—C2	−179.4 (2)	S1—C7—C9—C10	179.71 (16)
C7—S1—C1—C6	0.61 (15)	N1—C7—C9—C8	−173.65 (19)
C6—C1—C2—C3	1.2 (3)	S1—C7—C9—C8	4.7 (3)
S1—C1—C2—C3	−178.77 (17)	N2—N3—C10—C9	−6.8 (2)
C1—C2—C3—C4	0.4 (4)	C12—N3—C10—C9	−160.4 (2)
C2—C3—C4—C5	−1.3 (4)	N2—N3—C10—C11	171.05 (18)
C3—C4—C5—C6	0.5 (4)	C12—N3—C10—C11	17.4 (3)
C7—N1—C6—C5	−178.3 (2)	C8—C9—C10—N3	3.6 (2)
C7—N1—C6—C1	0.0 (3)	C7—C9—C10—N3	−172.02 (19)
C4—C5—C6—N1	179.4 (2)	C8—C9—C10—C11	−174.0 (2)
C4—C5—C6—C1	1.1 (4)	C7—C9—C10—C11	10.4 (4)
C2—C1—C6—N1	179.51 (19)	C8—N2—C13—C18	−74.5 (2)
S1—C1—C6—N1	−0.5 (2)	N3—N2—C13—C18	142.02 (19)
C2—C1—C6—C5	−2.0 (3)	C8—N2—C13—C14	101.9 (2)
S1—C1—C6—C5	177.98 (18)	N3—N2—C13—C14	−41.5 (3)
C6—N1—C7—C9	178.88 (19)	C18—C13—C14—C15	0.5 (3)
C6—N1—C7—S1	0.5 (2)	N2—C13—C14—C15	−175.8 (2)
C1—S1—C7—N1	−0.67 (16)	C13—C14—C15—C16	−0.2 (4)
C1—S1—C7—C9	−179.16 (17)	C14—C15—C16—C17	0.0 (4)
N3—N2—C8—O1	172.67 (19)	C15—C16—C17—C18	−0.2 (4)
C13—N2—C8—O1	25.2 (3)	C16—C17—C18—C13	0.5 (3)
N3—N2—C8—C9	−5.1 (2)	C14—C13—C18—C17	−0.7 (3)
C13—N2—C8—C9	−152.51 (18)	N2—C13—C18—C17	175.78 (19)
O1—C8—C9—C10	−176.5 (2)		
